# Neurofilament light chain (NfL) as a surrogate outcome measure for GM2 gangliosidoses

**DOI:** 10.1007/s00415-026-13984-x

**Published:** 2026-07-17

**Authors:** Kyriakos Martakis, Nicolas J. Abreu, Joshua J. Baker, Peter R. Baker II, Ian Billington, T. Andrew Burrow, Mallory Factor, Taylor Fields, Cassandra Fields, Jennifer L. Gannon, Megan Grosso, Jorgji Kerthi, Marc C. Patterson, Brian J. Shayota, Michael Strupp, Lennard Strupp, Tatiana Bremova-Ert

**Affiliations:** 1 Department of Pediatrics, University of Cologne, 50923 Cologne, Germany; 2 Department of Neuropediatrics, Justus Liebig University, Giessen, Germany; 3 Division of Neurogenetics, Department of Neurology, NYU Grossman School of Medicine, New York, NY USA; 4 Edwards Family Division of Genetics and Rare Diseases, Ann and Robert H Lurie Children’s Hospital of Chicago, Illinois, USA; 5 Department of Pediatrics, University of Colorado, Aurora, CO USA; 6 IntraBio Inc, Austin, TX USA; 7 College of Medicine, Department of Pediatrics, University of Arkansas for Medical Sciences, Little Rock, AR USA; 8 Department of Pharmacology, University of Oxford, Mansfield Road, Oxford, UK; 9 Division of Clinical Genetics, Children’s Mercy Hospital, Kansas City, MO USA; 10 Department of Pediatrics, University of Missouri-Kansas City School of Medicine, Kansas City, MO USA; 11 Division of Medical Genetics, Department of Pediatrics, University of Utah, Salt Lake City, UT USA; 12 Department of Neurology, Ludwig Maximilians University, Munich, Germany; 13 Department of Neurology, University Hospital Bern (Inselspital), Bern, Switzerland; 14 Third Department of Paediatrics, Medical School, Attikon’ University General Hospital, Chaidari, National and Kapodistrian University of Athens, Athens, Attica Greece

**Keywords:** GM2 gangliosidoses, Tay-Sachs disease, Sandhoff disease, Neurofilament light chain, Levacetylleucine, Biomarker

## Abstract

**Background:**

The GM2 gangliosidoses (GM2) are ultra-rare neurodegenerative disorders caused by deficient hexosaminidase A and/or B activity, leading to lysosomal GM2 ganglioside accumulation. Disease onset ranges from infancy to adulthood, with earlier onset associated with more rapid progression. Neurofilament light chain (NfL), a sensitive marker of axonal injury, has been extensively investigated as a biomarker for neurodegenerative disorders, including GM2.

**Methods:**

To evaluate its clinical utility as a biomarker for GM2, NfL was measured in patients with GM2 enrolled in a Phase 2b, multinational, rater-blinded study of levacetylleucine [NCT03759665], and in its open-label Extension Phase (EP).

**Results:**

Nineteen participants had viable samples for NfL analysis at baseline, after six weeks of treatment, and after a six-week washout; 10 had samples in the long-term EP. After the initial 6-week treatment phase, NfL concentration declined a mean − 8.9% (SD 13%; *p* < 0.008), followed by a rebound of + 9.2% (SD 16.1%; *p* = 0.022) during the post-treatment 6-week washout. Changes in NfL correlated with the statistically significant and clinically meaningful changes captured on the primary Clinical Impression of Change in Severity (CI-CS), and secondary Scale for the Assessment and Rating of Ataxia (SARA) and Modified Disability Rating Scale (mDRS). In the EP, patients showed a mean NfL reduction of − 16.9% after 1 year (SD 15.0; *p* = 0.010) and − 33.5% after 2 years (SD 12.8; *p* < 0.001) of levacetylleucine treatment.

**Conclusions:**

These findings support NfL as a promising surrogate outcome candidate for GM2 and link biochemical improvement with functional benefit, which is reasonably likely to predict both disease activity and treatment response/clinical benefit.

## Introduction

### Disease background

GM2 gangliosidoses (GM2) refer to progressive inherited neurodegenerative disorders caused by disease-causing variants in the *HEXA* or *HEXB* genes, which encode the alpha and beta subunits of β-hexosaminidase A, respectively, or in the GM2 activator gene (*GM2A*) that encodes the GM2 activator protein, a co-factor required for GM2 catabolism. Biallelic disease-causing variants in any of these 3 genes lead to storage of GM2 ganglioside in the central nervous system (CNS) with subsequent progressive neurodegeneration due to multiple processes such as apoptosis and inflammation causing synaptic loss and abnormal neuronal activity [1]. This results in a broad spectrum of neurological and psychiatric symptoms and premature death.

GM2 comprises a family of ultra-rare diseases (Tay-Sachs disease, Sandhoff disease, and AB Activator Variant), for which there is currently no approved disease-modifying therapy (in the United States or worldwide). Ultra-rare diseases have been defined as those affecting fewer than 1:50,000 people, or approximately 7000 people in the United States [2, 3]. Comparably, the global prevalences of Tay-Sachs and Sandhoff diseases have been estimated as 1 in 417,000–588,000 and 1 in 500,000–1,500,000 individuals, respectively [4, 5]. In terms of clinical trials, it has been recognized that within the ultra-rare disease rubric, many entities have so few patients that traditional trial designs and statistical methods are not feasible [6].

### NfL as a biomarker in neurodegenerative disorders

Neurofilament light chain (NfL) is a neuronal cytoskeletal protein which is highly expressed in large caliber myelinated axons, where it forms part of the neuroaxonal scaffold [7]. Axonal degeneration or injury is a predominant feature of many neurodegenerative disorders that lead to most often irreversible impairment. In response to such damage, NfL is released into the extracellular space. Consequently, elevated NfL concentrations in the cerebrospinal fluid (CSF) and blood are observed in most neurodegenerative disorders, along with inflammatory, traumatic, and vascular conditions [8–11]. NfL expression is largely restricted to the CNS. NfL in plasma strongly correlates with its levels in the CSF in pathological states [12–14].

NfL has been extensively investigated as a biomarker in a wide range of neurological disorders, including neurodegenerative disorders [15–19] as well as ataxias [20–22]. Specific disorders include frontotemporal dementia (FTD) [23, 24], Parkinson disease and related synucleinopathies [25, 26], amyotrophic lateral sclerosis (ALS) [27–30], immune-mediated disorders (multiple sclerosis [MS]) [31, 32], epilepsy [33], and trauma [34]. NfL concentrations have been shown to be elevated in carriers of pathogenic variants predicting symptom onset in genetic forms of Huntington Disease [35], ALS [28], and FTD [36].

The concentration of NfL can be measured in both CSF and blood using single molecule arrays (e.g., Simoa—Quanterix, Billerica, MA, USA) or microfluidic assays (e.g., Ella-Bio-Techne, Minneapolis, MN, USA) [37]. Spurred by the recent availability of such sensitive assay technology, plasma NfL is increasingly finding utility as both a monitoring and prognostic biomarker of neuronal damage and degeneration in a variety of disorders [7, 38]. For instance, in MS, plasma NfL is elevated, correlates with magnetic resonance imaging (MRI) measured lesions, and is reduced by approved treatments [13, 39, 40]. In ALS, NfL has been established as a sensitive and valid biomarker of axonal injury and neurodegeneration, which correlates with the speed and severity of ALS progression. In this disease, NfL has been accepted as a valid surrogate endpoint reasonably likely to predict clinical benefit. Reductions of plasma NfL during treatment with tofersen (QALSODY®) were also the basis for its accelerated approval for the therapy of ALS in adults who have a pathogenic variant in the superoxide dismutase 1 (*SOD1*) gene [41, 42].

### NfL in healthy individuals

In healthy individuals, concentrations of NfL have been demonstrated to increase with age, consistent with the natural process of aging. In recognition of the growing support of the use of plasma NfL as a biomarker for the evaluation of potential disease-modifying treatments, reliable cut-offs were elaborated by pooling quantified plasma NfL in neurologically healthy participants (5–90 years) using the Simoa assay [43]. An independent study measured serum NfL concentrations in a cohort of 2667 healthy children under 18 years of age using the same assay [44]. In healthy children, NfL was transiently very high after birth, likely related to relatively low blood volume in the first years of life. It decreased by 6.8% every year until the age of 10 and then remained mostly stable up to the age of 22 years. Independent of age, the magnitude of the effect of weight on serum NfL concentrations was marginal. The average NfL for a healthy child was 4.8 pg/mL.

### NfL as a biomarker for GM2 and other lysosomal storage disorders (LSDs)

In vitro and in vivo studies in models of GM2 as well as studies in patients have established the utility of NfL as a biomarker for this disorder [45–47]. Similarly, NfL has been established as a potential biomarker for other related LSDs [48, 49].

### Findings from GM2 mouse model

The Hexb-/- mouse model of Sandhoff disease closely recapitulates human infantile-onset GM2, exhibiting early neurological signs by 6–8 weeks, progressive motor deficits (comparable with patient’s severe motor and developmental impairments), and a markedly shortened lifespan of approximately 16–18 weeks (consistent with the median age of death of 4 years in patients) [47]. Multiple independent preclinical experiments in this model have established plasma NfL as a reliable biomarker for monitoring disease progression and treatment response, consistently showing that elevated NfL in untreated Hexb-/- mice correlates with clinical deterioration, and therapeutic intervention normalizes NfL concentrations toward those of wild-type controls.

Two lines of preclinical evidence reinforce this relationship. First, a clear dose–response correlation was demonstrated with sinbaglustat, wherein higher drug exposure produced greater reductions in plasma NfL alongside improved motor performance and extended survival [45, 46]. Second, it was shown that bone marrow transplant, alone or combined with colony-stimulating factor 1 receptor (CSF1R) inhibition, substantially reduced NfL concentrations relative to untreated Hexb-/- mice, with corresponding functional improvements [47]. Across these studies, reductions in plasma NfL consistently corresponded with slower disease progression and better outcomes, supporting its utility as a meaningful translational biomarker in GM2.

### NfL in human GM2

In humans it was found that plasma NfL concentrations were markedly elevated in all 33 GM2 patients studied, both Tay-Sachs and Sandhoff, with no overlap with the 66 healthy controls [45] which also confirms NfL’s sensitivity as a marker of neuroaxonal injury in this disease. NfL concentrations correlated strongly with clinical severity and age of onset, with infantile-onset patients showing median levels 42.5-fold above controls and 13-fold and 4.3-fold elevations in juvenile and adolescent-onset patients, respectively, a gradient consistent with the underlying enzyme activity and established phenotypic classifications. Notably, the inverse correlation between age of onset and NfL plasma concentration is consistent with the correlation between clinical severity and age of onset in GM2 patients, which also correlates to the level of enzyme activity (of the β-hexosaminidase A, β subunit, or GM2 ganglioside activator). Collectively, these findings position plasma NfL as a robust, clinically meaningful biomarker capable of both distinguishing GM2 patients from healthy individuals and stratifying disease burden across the spectrum of GM2.

### Findings in other LSDs

In other studies, it was demonstrated that plasma NfL is also elevated in children with neuronal ceroid lipofuscinosis type 2 (CLN2) [48] and neuronopathic mucopolysaccharidosis type II [49]. CLN2 patients receiving intracerebroventricular infusions of the enzyme replacement therapy cerliponase alfa, which attenuated the rate of motor decline, showed reduction in plasma NfL of 50% per year over a 3-year treatment period [50]. Similarly, NfL concentrations have been shown to correspond with the degree of primary substrate burden and clinical outcomes in patients with neuronopathic mucopolysaccharidosis type II [51]. Elevated NfL, particularly when combined with glucosylsphingosine levels and abnormal auditory brainstem response, distinguishes neuronopathic Gaucher disease with neurological involvement from non-neuronopathic forms and enables earlier identification of severe disease even before symptoms appear [52].

### Summary of studies in animals and humans

Recent advancements demonstrate that NfL is a non-specific, but robust, marker of axonal injury in many neurodegenerative diseases, including GM2. The *Hexb*^*−/−*^ mouse, an established model of GM2, shows elevated concentrations of NfL. Two studies with different investigational treatments have shown dose-dependent relationships between lower plasma NfL and improvements in symptoms, supporting NfL as a valuable biomarker for the evaluation of treatment effect in GM2. In GM2 patients, NfL concentrations are clearly elevated and do not overlap with controls. A direct correlation can also be established between NfL plasma level and age of disease onset, which is also associated with the level of enzyme activity in GM2, the basis and key driver of the disease. NfL concentrations do not increase with age in pediatric and young adult controls. NfL concentrations in other neurodegenerative LSDs, including NPC [53], metachromatic leukodystrophy [54], CLN2, ceroid-lipofuscinosis type 3 (CLN3) [55], and neuronopathic mucopolysaccharidosis type II, also correspond to disease activity and decrease in response to treatment.

### Levacetylleucine

Levacetylleucine (N-acetyl-L-leucine) is a modified, acetylated derivative of a natural essential amino acid (L-Leucine). It is orally administered and transported via monocarboxylate transporters, which are ubiquitously expressed, thereby delivering levacetylleucine to all tissues and across the blood–brain barrier to the CNS, delivering high levels of levacetylleucine relative to leucine into the cytoplasm [56]. Inside cells, levacetylleucine enters enzyme-controlled pathways that correct metabolic dysfunction, including ameliorating dysfunction of the lysosomal-mitochondrial axis, improving energy (adenosine triphosphate [ATP]) production and normalizing lysosomal function [57, 58]. The knock-on effects of enhancing mitochondrial and lysosomal health include reducing neuroinflammation and improving cellular function (e.g., normalizing neuronal membrane potential and thereby intercellular signaling) [57, 59–62] leading to an overall restoration of neuronal function and preventing/protecting against neurodegeneration.

The efficacy of levacetylleucine for GM2 has been previously demonstrated in vitro, in vivo, and clinical studies [57, 63–65]. In the GM2 mouse model (*Hexb*^*−/−*^*),* acetylleucine was found to significantly delay the onset of functional decline (gait abnormalities, motor dysfunction), the decline in general health and condition, and slowed disease progression, prolonging survival [57]; these symptomatic and disease-modifying effects have also been demonstrated in observational clinical studies [57, 64].

Based on these positive findings, a multinational, rater-blinded Phase 2b clinical trial of levacetylleucine for pediatric and adult patients with GM2 was conducted [NCT03759665] and demonstrated a statistically significant and clinically meaningful improvement in symptoms, functioning, and quality of life for children and adults with GM2 after 6 weeks of treatment [65]. Similar positive studies in the related LSD Niemann-Pick disease type C (NPC) have been conducted, also demonstrating levacetylleucine significantly improves neurological manifestations, and has a disease-modifying effect [66–68]. In all studies, levacetylleucine has exhibited a benign and well-tolerated safety profile with no serious side effects. Currently, levacetylleucine (AQNEURSA®) is authorized in the United States and the European Union for the treatment of neurological manifestations in NPC [69, 70].

Based on the positive preclinical and clinical findings with levacetylleucine for GM2, in this study, we aimed to investigate the utility of NfL as a biomarker for GM2, including its utility to predict disease activity and reflect treatment response/clinical benefit.

## Methods

### IB1001-202 parent study

NfL samples were analyzed from participants of the Phase 2b trial with levacetylleucine for GM2 (study IB1001-202, NCT03759665) [6]. This was a rater-blinded, open-label Phase 2b study conducted at 6 sites in Germany, Spain, the United Kingdom (UK), and the United States (US) in patients with a confirmed genetic diagnosis of GM2. Adults and children aged 6 years and older with a confirmed genetic diagnosis of GM2 gangliosidoses were eligible to participate; patients using prohibited medications at screening (i.e., medications that may have confounded the safety or efficacy analysis of the trial, including N-acetyl-dl-leucine, N-acetyl-L-leucine, or aminopyridines [prohibited if not provided as the investigational medicinal product], varenicline, riluzole, sulfasalazine, chlorzoxazone, gabapentin, or rosuvastatin) were required to complete a 42-day washout prior to their first baseline visit. The eligibility criteria were previously published [6].

The IB1001-202 trial was conducted in accordance with the International Conference for Harmonisation (of Technical Requirements for Pharmaceuticals for Human Use), Good Clinical Practice Guideline, the General Data Protection Regulator, and the Declaration of Helsinki. The trial design, including rationale, methodology, and study results has been previously published [6]. Written informed consent was obtained for all study participants by the patient or, if applicable, their parent or legal representative.

**Fig. 1:**
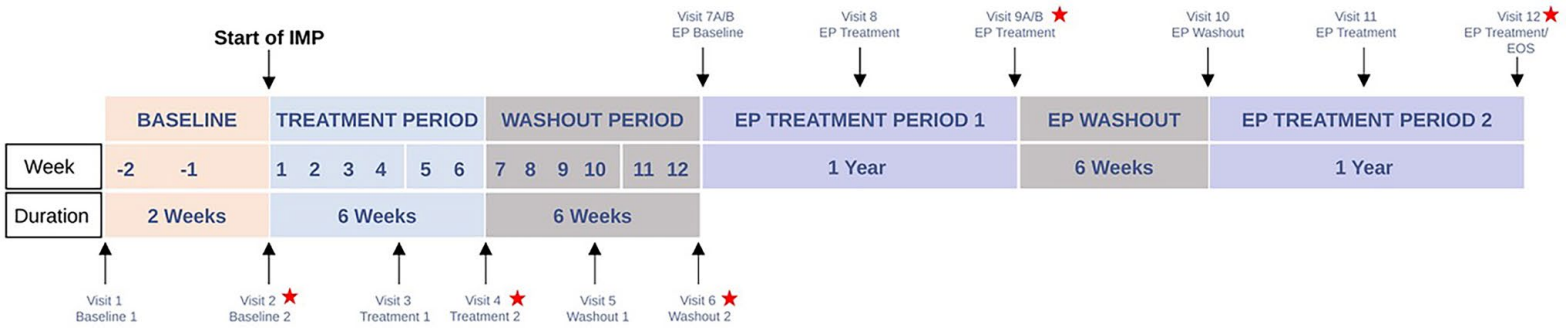
IB1001-202 Parent Study and extension phase design. *EP* extension phase, *IMP* investigational medicinal product. Red start indicates visits at which NfL samples were collected

In the “Parent Study”, patients were assessed during three consecutive study periods: a 2-week (+ 7 day) baseline period, a 6-week (+ 7 day) treatment period (in which all patients were to receive levacetylleucine, and a 6-week (+ 7 day) post-treatment washout period (Fig. 1). The primary endpoint, the Clinical Impression of Change in Severity (CI-CS), was based on patient’s performance on either the 9 Hole Peg Test–Dominant Hand (9HPT-D), or the 8-Meter Walk Test (8MWT) was compared based on video recordings taken at baseline (visit 2), the end of treatment (visit 4), and the end of the washout period (visit 6). For each patient, either the 9HPT-D or 8MWT was chosen as the primary assessment measure by the principal investigator at visit 1, based on their unique individual symptoms. Sites were trained on a standardized protocol to ensure that the 9HPT-D and 8MWT were filmed consistently, and the videos were uploaded to be centralized assessed by a team of 3 certified neurologists. Two of these neurologists reviewed randomized, blinded video pairs as follows: baseline vs end of treatment (pair A), end of treatment vs end of washout (pair B), and baseline vs end of washout (pair C). For each pair, the raters had to assess the change of severity of the patient’s signs using a 7-point Likert scale. The third rater acted as an adjudicator, when the results of the assessment of the 2 primary raters differed by more than 1 point on the Likert scale. The CI-CS was defined as the change from pair A minus pair B; thus, the washout period served as the control arm to the treatment period [6].

Secondary endpoints included the Scale for the Assessment and Rating of Ataxia (SARA), an eight-item clinical rating scale that incorporates assessments of gait, stance, sitting, and speech disturbance, as well as the finger-chase test, the nose-to-finger test, the fast-alternating-hand- movements test, and the heel-along-shin slide test ranging from 0 (best) to 40 (worst) [71] and the Modified Disability Rating Scale (mDRS), a measurement of overall neurologic status which consists of six subdomains (ambulation, manipulation, seizures, language, swallowing, and ocular movements), with the total score for overall neurologic status ranging from 0 (best) from 24 (worst); scores were then scaled to a range of 0 to 1 [72].

### IB1001-202 Extension Phase (EP)

In the EP, patients were assessed approximately 6 times over a 116-week period, receiving treatment with levacetylleucine for approximately 2 years [6].

### Analysis of NfL plasma concentrations

During Study IB1001-202, blood samples for research purposes were obtained at Visit 2 (baseline), Visit 4 (after 6 weeks’ treatment with levacetylleucine), and Visit 6 (after 6 weeks’ washout) of the Parent Study, and at Visit 9 and Visit 12 of the EP. NfL concentrations were analyzed using a validated assay at Medpace Reference Laboratories. The analyzed set contained 97 samples collected between 28-Jun-2019 through 09-Jan-2023 from 30 patients enrolled in IB1001-202.

Ninety-seven samples were received at Medpace Reference Laboratories frozen and in good condition. NfL concentrations were assayed using the Simoa® Human Neurology 4-Plex E (N4PE +) Advantage Plus Assay on the HD-X platform [73].

No test–retest reliability assessment of the NfL samples was planned as part of the study, but five back-up blood samples from Visit 9 were included in the analyzed set and were compared to the primary samples. The inter-sample variation had a mean of 2.9% and range of 0 to 5.7%, which was within the 7.2 to 10.3% coefficient of variation (CV) cited in the assay data sheet [73].

### Statistical methods

This was an analysis of plasma samples collected for research purposes as part of Study IB1001-202. No adjustments were made for multiple comparisons.

Baseline NfL concentrations are presented for all 27 patients with NfL measurements at Visit 2. The Parent Study NfL analysis set consisted of 19 patients with reliable, recorded NfL measurements at each of Visits 2 (baseline – the start of levacetylleucine treatment), Visit 4 (after approximately 6 weeks levacetylleucine treatment), and Visit 6 (after approximately 6-week post-treatment washout from levacetylleucine) during the Parent Study and with samples defined as being reliably obtained and processed. For these patients, the change from baseline and percentage change from baseline at each visit were calculated. Change during the Washout Period (Visit 4 to Visit 6) was also calculated.

The EP NfL analysis set consisted of 10 patients who recorded NfL concentration values at either or both of Visit 9 and Visit 12. One of the 10 patients did not record a value at Visit 9, and 3 patients did not record a value at Visit 12. For both the Parent Study and the EP visits, 1-sample t-tests were used to test the null hypotheses of no change in NfL concentrations from baseline and no change in NfL concentration during the Washout Period. For the Parent Study population, the change in each of the SARA, CI-CS, and mDRS clinical endpoints during the Treatment Period and Washout Period were compared to the percentage change in NfL concentration during the corresponding periods. Pearson *r* between the change in each clinical scale and percentage change in NfL concentration was computed.

A statistical comparison of NfL concentrations to the SARA and mDRS clinical scales was made by regressing percentage changes in NfL concentration against change in clinical scales using Ordinary Least Squares (OLS) implemented in statsmodels [74]. The CI-CS is an ordinal scale, so percentage changes in NfL concentration were fitted to CI-CS values with half-point intervals between -3 and + 3 using the ordered model implemented in statsmodels.

## Results

### Summary of clinical findings from IB1001-202

#### Parent study

In total, thirty-six participants were screened between June 7, 2019, and October 1, 2020, and 30 patients qualified for inclusion (including 27 Tay-Sachs patients and 3 Sandhoff patients). Patient baseline demographic and clinical characteristics are shown in Table 1A. The findings from the Parent Study were that levacetylleucine rapidly improved neurological signs, symptoms, functioning, and quality of life after 6 weeks of treatment, followed by a deterioration (return to baseline status) in the 6-week post-treatment washout period [65]. The study met its primary Clinical Impression of Change in Severity (CI-CS) endpoint; Hodges-Lehmann estimator was 0.75 (90%CI 0.00, 1.50; *p* = 0.044). Pre-defined subgroup analyses based on age, disease severity, age of symptom onset, etc. did not show noteworthy differences (taking into account the small sample size of some subgroups), which might indicate levacetylleucine’s therapeutic applicability for all GM2 patients. A high level of consistency between the primary and secondary endpoints further reinforced the clinically meaningful effects of levacetylleucine. The analysis of Scale for the Assessment and Rating of Ataxia (SARA) total score indicated improvement in cerebellar signs and symptoms at the end of treatment compared with baseline (Hodges-Lehmann estimator -1.25; 90%CI: − 1.75, − 0.75; *p* < 0.001) and deterioration in cerebellar signs and symptoms during washout (Hodges-Lehmann estimator 1.25; 90%CI 0.50, 2.00; *p* = 0.001). The analysis of the Modified Disability Rating Scale (mDRS) composite score also indicated neurological improvement at the end of treatment compared with baseline (Hodges–Lehmann estimator − 0.031; 90%CI: − 0.063, 0.000;* p* = 0.020) and neurological deterioration during washout (Hodges–Lehmann estimator 0.042 (90%CI 0.021, 0.063; *p* < 0.001), with 62% of EP patients achieving no worsening or improvement on mDRS. The physician, caregiver, and patient Clinical Global Impression of Change (CGI-C) scores were consistent and supported an overall improvement in patients’ overall symptoms, functioning, and condition during the treatment period and deterioration during the washout period.

**Table 1:** Baseline characteristics and demographics from study IB1001-202 [65]

(A) Parent study		
Age (years)	Mean (SD)	27.0 (15.2)
	Median	28.5
	Range	6.0–55.0
Gender, *n* (%)	Male	11 (36.7%)
	Female	19 (63.3%)
Age group, *n* (%)	Pediatric (< 18 years)	10 (33.3%)
	Adult (≥ 18 years)	20 (66.7%)
Dose, *n* (%)	Age 6–12 years—15 to < 25 kg—2 g per day	3 (10.0%)
	Age 6–12 years—25 to < 35 kg—3 g per day	4 (13.3%)
	Age 6–12 years— ≥ 35 kg—4 g per day	1 (3.3%)
	Age ≥ 13 years—4 g per day	22 (73.3%)
Disease, *n* (%)	Tay-Sachs	27 (90.0%)
	Sandhoff	3 (10.0%)
(B) Extension phase		
Age (years)	Mean (SD)	18.6 (12.1)
	Median	13.0
	Range	6-38
Gender (*n* (%))	Male	5 (38.5%)
	Female	8 (61.5%)
Age group (*n* (%))	Pediatric (< 18 years)	7 (53.8%)
	Adult (≥ 18 years)	6 (46.2%)
Dose group (*n* (%))	Age 6–12 years—15 to < 25 kg—2 g per day	2 (15.4%)
	Age 6–12 years—25 to < 35 kg—3 g per day	4 (30.8%)
	Age ≥ 13 years—4 g per day	7 (53.8%)
Disease (*n* (%))	Tay-Sachs	12 (92.3%)
	Sandhoff	1 (7.7%)

### Extension phase

Fourteen of the 27 patients who completed the Parent Study (Visit 6) entered the EP (Patient baseline demographic and clinical characteristics are shown in Table 1B). The baseline visit occurred at or after the last visit of the Parent Study, after each patient had completed a minimum 6-week washout from levacetylleucine. The primary endpoint for measuring long-term efficacy was the mDRS score. In IB1001-202, the proportion of patients with success (defined as no change or an improvement) on the mDRS score (Visit 9 versus Visit 7; Treatment Period I) was 0.62 (90%CI 0.38, 0.81) and reached statistical significance with *p* < 0.001 as compared to a proportion of 0.10 for the modified intention-to-treat EP (mITTe) population.

### NfL values at baseline in study IB1001-202

**Fig. 2:**
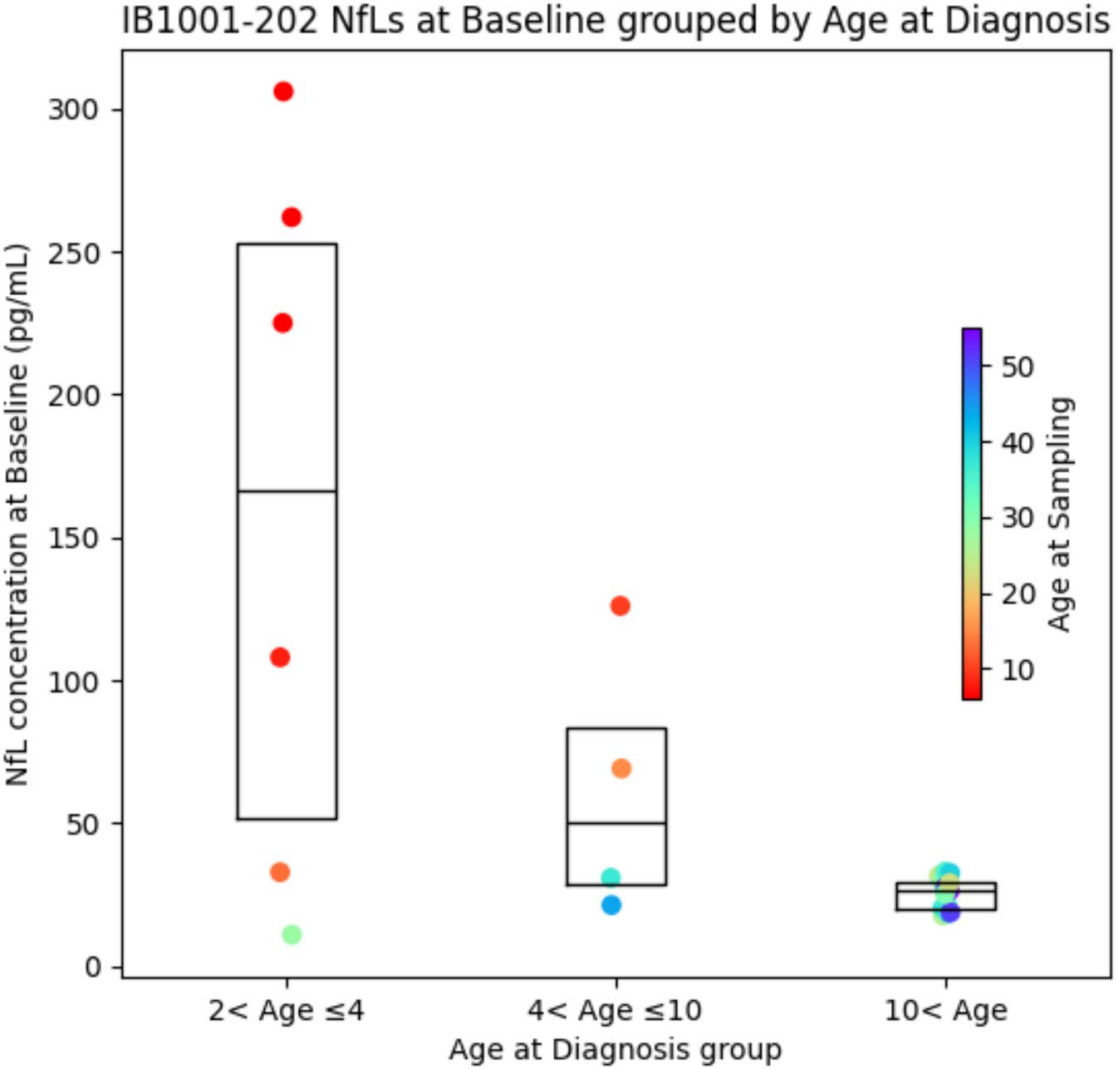
Plasma NfL concentration at baseline of the IB1001-202 cohort, grouped by age at diagnosis. N = 27. Includes all patients from Study IB1001-202 with a baseline (i.e., Visit 2) NfL measurement. Boxes show median and inter-quartile ranges by Age at Diagnosis group. Individual patient concentrations are superimposed with points colored by age at sampling

NfL assays were obtained from 27 individual participants with GM2 during the Parent Study. At baseline (Visit 2), the cohort showed elevated concentrations of NfL in comparison to the corresponding age group at diagnosis as reported by Simrén et al. (2022) [43] and Welford et al. (2022) [45]. Six patients with age of diagnosis between 2 and 4 had a mean baseline (SD) NfL concentration of 157.1 pg/mL (124.1); four patients with age of diagnosis between 4 and 10 had a mean baseline NfL concentration of 61.8 pg/mL (47.5); and seventeen patients aged over 10 at diagnosis had a mean baseline concentration of 25.7 pg/mL (5.3) (see Fig. 2).

### NfL values by visit in study IB1001-202 parent study

**Fig. 3:**
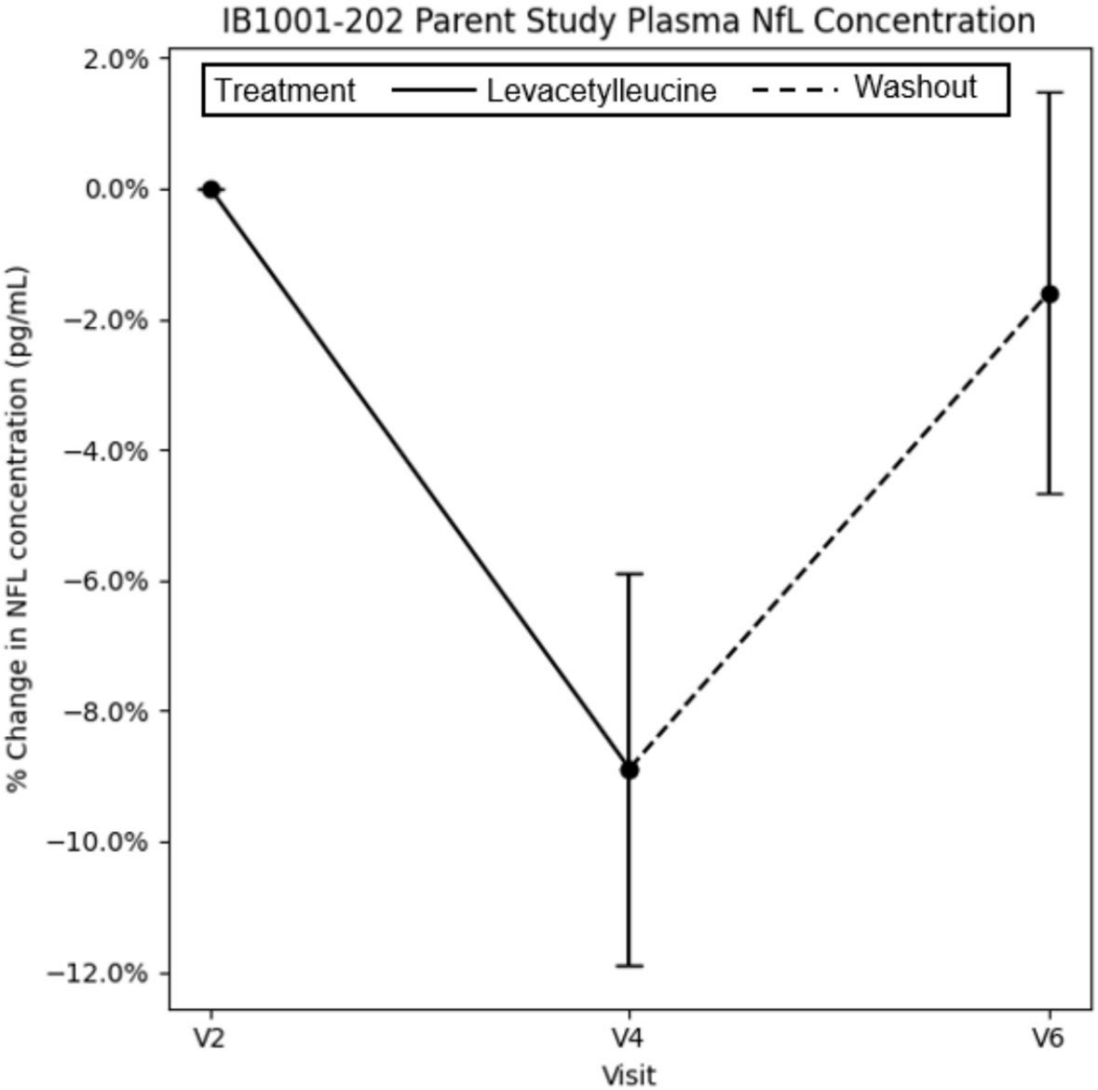
Change in NfL values in the Study IB1001-202 Parent Study (Parent Study NfL analysis set). *V2* (baseline visit): 0 months; *V4* 6-week levacetylleucine treatment; *V6* 6-week washout from levacetylleucine [12 weeks post-baseline]. Solid line represents levacetylleucine treatment, dashed line represents post-treatment washout

Nineteen patients with NfL assays at Visit 2, Visit 4, and Visit 6 were included in the analysis set. These patients showed a statistically significant mean reduction in NfL concentrations following 6 weeks’ administration of levacetylleucine (Visit 2 to Visit 4) (mean [SD] − 8.9% [13.1%], *p* = 0.008), which subsequently rebounded during the 6-week washout period (Visit 4 to Visit 6) (+ 9.2% [16.1%], *p* = 0.022) (Table 2). Owing to the wide range of absolute values for NfL (reflecting the varying ages of onset in the study cohort), the findings are displayed as percentage change in the NfL concentration (Fig. 3).

**Table 2:** Change from baseline in NfL values in the study IB1001-202 Parent Study (Parent Study NfL analysis set)

Visit	Timepoint evaluated	N	Mean (SD)	Mean (SD) % Change from Baseline (Visit 2)	95% CI	*p* value	Mean (SD) % Change from levacetylleucine Treatment (Visit 4)	95%CI	*p* value
Visit 2	Baseline	19	70.4 pg/mL (89.8)						
Visit 4	End of 6-week levacetylleucine treatment	19	62.9 pg/mL (79.3)	− 8.9% (13.1%)	− 15.2% to − 2.6%	0.008			
Visit 6	End of 6-week post-washout from levacetylleucine	19	68.3 pg/mL (86.9)	− 1.6% (13.4%)	− 8.0% to 4.9%	0.610	9.2% (16.1%)	1.5% to 17.0%	0.022

Visit 2: baseline; Visit 4: end of 6-week levacetylleucine treatment; Visit 6: end of 6-week post-washout from levacetylleucine

These descriptive statistics were also computed for the subsets of “juvenile” patients (aged ≤ 10 at baseline) and “adolescent and adult” patients (aged > 10 at baseline). Patients whose age at baseline was ≤ 10 showed a mean [SD] change from baseline of − 12.6% [3.7%] following administration of levacetylleucine, and then rebounded + 10.7% [5.1%] during the six-week washout period. The subset of “adolescent and adult” patients whose age was > 10 at baseline showed a mean [SD] change from baseline of − 7.9% [14.6%] following administration of levacetylleucine and then rebounded + 8.8% [18.1%] change from baseline after the six-week washout period.

**Fig. 4:**
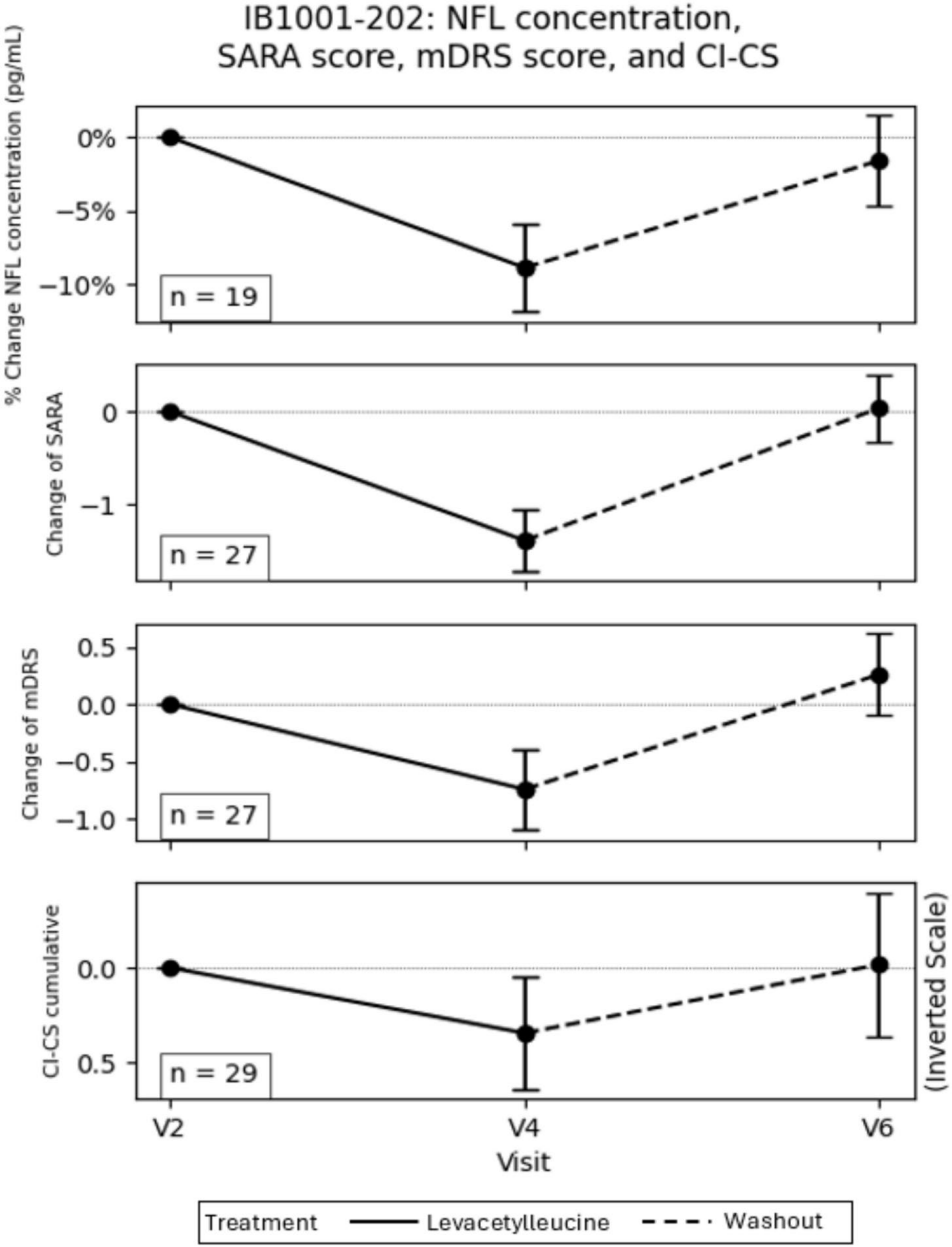
Parallel Changes in NfL concentration, SARA, mDRS, and CI-CS by treatment period. Changes in the CI-CS primary endpoint, the SARA scale and mDRS secondary endpoints, and NfL concentration during the IB1001-202 Parent Study. For SARA and for mDRS a negative change represents an improvement. For CI-CS a positive change is an improvement, so it is plotted on an inverted y-axis to align the changes with SARA

The observed changes in NfL concentrations paralleled the improvement observed in clinical outcome measures during the treatment and washout phases (Fig. 4).

### Observed correlation between NfL concentrations and clinical outcomes

The percentage change in patients’ plasma NfL concentration was compared to changes in total SARA score, mDRS, and CI-CS (Table 3). The periodic changes in each scale were computed for the treatment period and for the Washout Period of the IB1001-202 Parent Study. Ordinary Least Squares (OLS) was then used to find a best fit model of change in SARA and mDRS as a function of percentage change in NfL concentration. An Ordered Model was used to compare changes in NfL with CI-CS.

**Table 3:** Statistical analysis of changes in NfL concentration and changes in efficacy outcome measures in IB1001-202 Parent Study (Parent Study NfL analysis set**)**

SARA (*n* = 19)	Pearson *r*	0.38			
	OLS	Coefficient	Standard error	95%CI	Two-sided *p* value
	m^a^	5.4	2.1	1.2, 9.6	0.013
	c^b^	0.0	0.4	− 0.7, 0.7	0.939
mDRS (*n* = 19)	Pearson *r*	0.30			
	OLS	Coefficient	Standard error	95%CI	Two-sided *p* value
	m	3.2	1.6	− 0.1, 6.4	0.056
	c	− 0.1	0.3	− 0.6, 0.5	0.788
CI-CS (*n* = 19)	Pearson *r*	0.18			
	Ordered Model	Coefficient	Standard error	95% CI	Two-sided *p* value
	NfL^c^	− 2.2	1.7	− 5.5, 1.1	0.184

^a,b^m is the linear coefficient and c the intercept for a regression model: *Δclinical-endpoint* = *m* × *ΔNFL%* + *c*

^c^NfL is the latent coefficient of Δ NFL% for the Ordered Model

*OLS* ordinary least squares

The comparison between percentage changes in NfLs (Δ NFL%) and changes in points in SARA scores (Δ SARA) provided a Pearson *r* between changes in the two scales of 0.38. The linear slope term linking the two endpoints was m = 5.4 (95%CI 1.2, 9.6), *p* = 0.013, and the intercept was at the origin c = 0.0 (95%CI − 0.7, + 0.7) indicating a statistically significant linear relationship between changes in the SARA scale and NfL concentrations.

The Pearson *r* between percentage change in NfL concentration and change in mDRS was 0.30. The best fit OLS model slope term was *m* = 3.2 (95%CI − 0.1, 6.4), *p* = 0.056 and the intercept was *c* = − 0.1 (95% CI -0.6, + 0.5).

The Pearson *r* between CI-CS and percentage change in NfL was 0.18 reflecting that a positive CI-CS representing an improvement in symptoms was correlated with a reduction in NfL concentration. The Ordered Model regression coefficient of CI-CS against percentage change in NfL, − 2.2 (95%CI − 5.5, 1.1) also indicating a relationship between an improvement in symptoms and a reduction in NfL concentration that did not reach statistical significance.

### NfL values in the study IB1001-202 EP

NfL data at Visit 9 and/or Visit 12 are available for 10 study participants. Percentage change from baseline (Visit 2) to visits in the Parent Study and the long-term EP is presented in Table 4 (note, Visit 6 represents a washout from drug).

**Table 4:** Percentage change in NfL values of patients who participated in both the Parent Study and EP (EP NfL analysis set)

Visit	Timepoint evaluated	*n*	Mean NfL concentration (SD)	Mean % Change from baseline (SD)*	CI	*p* value (vs no change from baseline)
Visit 4	End of 6-week levacetylleucine treatment	8	125.8 (99.4)	− 10.2% (8.6%)	− 17.4%, -3.0%	0.012
Visit 6	End of 6-week post-washout from levacetylleucine	8	125.0 (113.4)	2.1% (10.2%)	− 6.4%, 10.7%	0.573
Visit 9**	Approximately 15 months post-baseline, 1-year levacetylleucine treatment	9	96.6 (85.1)	− 16.9% (14.9%)	− 28.3%, − 5.4%	0.010
Visit 12	Approximately 27 months post-baseline, 2-year levacetylleucine treatment	7	70.8 (56.9)	− 30.5% (12.8%)	− 42.3%, − 18.7%	< 0.001

*Baseline value is Visit 2 value. Percentage change from Baseline was computed per patient per visit. Mean (SD) percentage change was computed from only patients completing a given visit

**Note: For Visit 9, V9BPOB measurement values were utilized in the NfL analyses because this was the larger of the two sample datasets across the EP patients

Patient samples demonstrated a statistically significant average reduction in NfL concentrations following 1-year administration of levacetylleucine (Visit 2 to Visit 9): -16.9% (SD = 15.0),* p* = 0.010, and a further substantial reduction following two years of treatment with levacetylleucine (Visit 2 to Visit 12): -30.5% (SD = 12.8%), *p* < 0.001 (Table 4).

These NfL assays on samples obtained during the EP showed a significant progressive decline in NfL status, indicative of improvements in neuroaxonal damage and an amelioration of neural cell death, and consistent with the clinical findings from the EP.

**Fig. 5:**
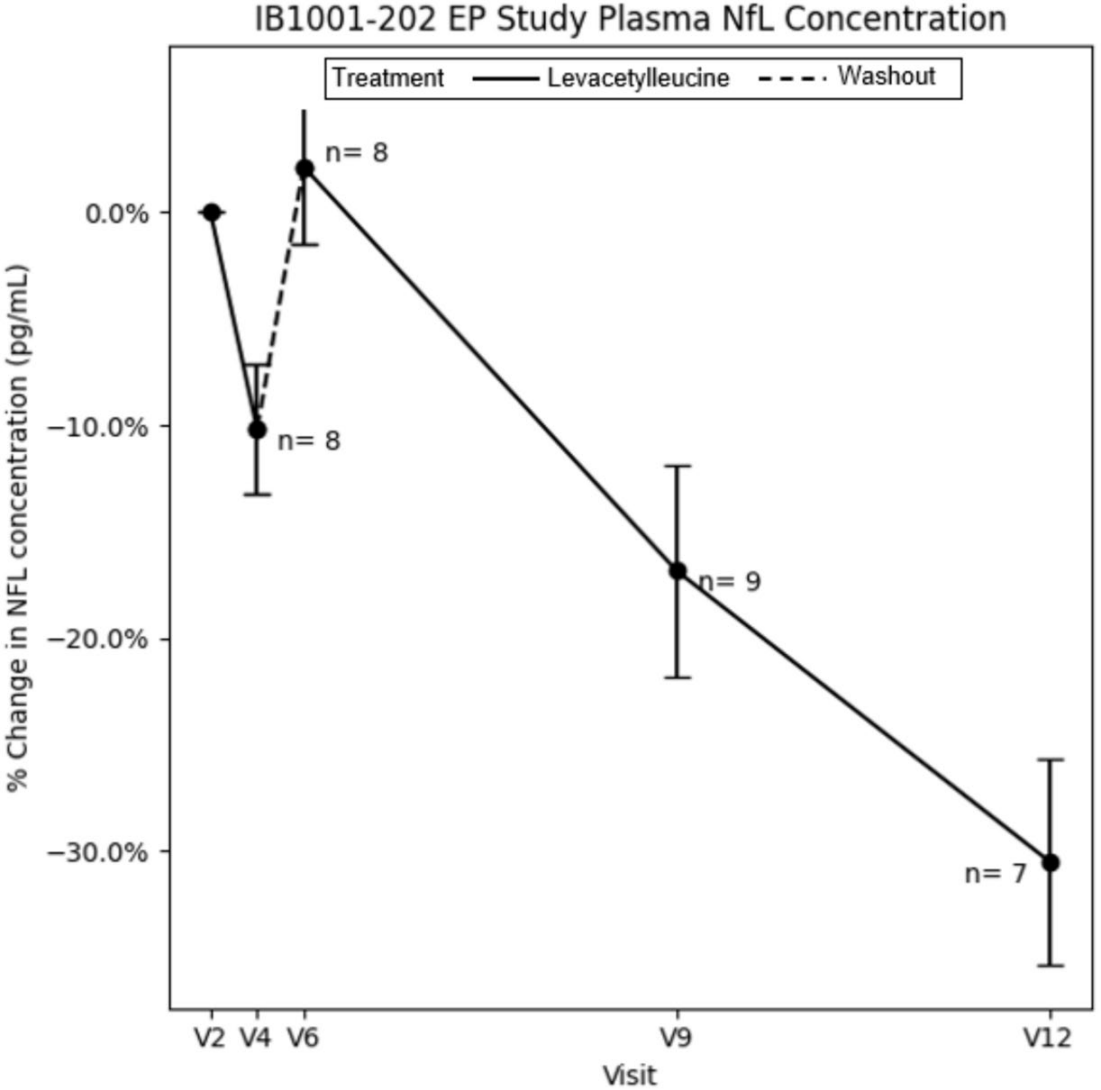
Mean (+ / − SE) % change in plasma NfL concentration during the Parent Study (Visits 2, 4 and 6) and EP (Visits 9 and 12) of the IB1001-202 trial (EP NfL analysis set). *V2* baseline visit; *V4* 6 weeks levacetylleucine Treatment; *V6* 6 weeks washout from levacetylleucine [12 weeks post-baseline]; *V9* 12-month levacetylleucine treatment [15-month post-baseline]; *V12* 24-month levacetylleucine treatment [27-month post-baseline]

**Fig. 6:**
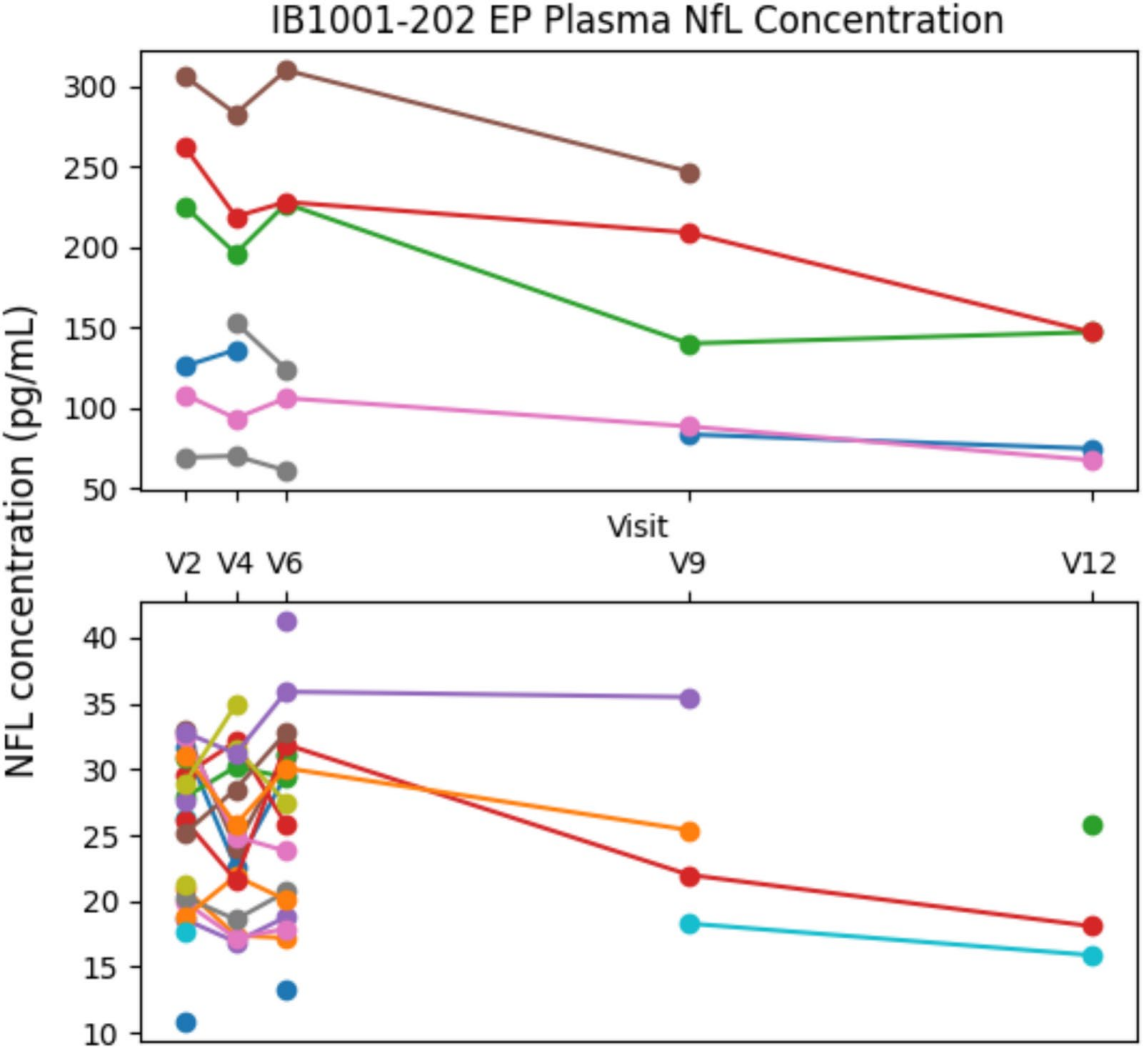
Individual NfL concentrations per patient during the Parent Study (Visits 2, 4, and 6) and EP (Visits 9 and 12) (EP NfL analysis set). Samples obtained at sequential visits are shown as values with a connected line. Samples obtained at discontinuous visits noted as solitary dots. *V2* baseline visit; *V4* 6 weeks levacetylleucine Treatment; *V6* 6 weeks washout from levacetylleucine [12 weeks post-baseline]; *V9* 12-month levacetylleucine treatment [15-month post-baseline]; *V12* 24-month levacetylleucine treatment [27-month post-baseline]

Mean (SE) NfL concentrations for the Parent Study and EP are displayed in Fig. 5. NfL concentrations for individual patients in the Parent Study and the EP are displayed in Fig. 6.

## Discussion

These data from a Phase 2b, multinational, rater-blinded study of levacetylleucine (Parent Study), and in its open-label Extension Phase in patients with GM2 show that NfL concentration changes correlate with clinical outcome measures, and therefore a potential surrogate outcome measure. In the United States, a surrogate biomarker used to support regulatory approval is generally defined as a laboratory measurement, radiographic image, physical sign, or other measure that is reasonably likely to predict a clinically meaningful outcome, but is not itself a measure of clinical benefit [75]. The major finding from this analysis is that NfL concentrations are a robust marker of axonal injury which are consistently elevated above age-related control levels in GM2, that do not fall spontaneously, and which have been shown in a murine model of GM2 to correlate with drug exposure and therapeutic response indeed corresponded with drug exposure and therapeutic response in the IB1001-202 clinical trial of pediatric and adult patients with patients with GM2 gangliosidoses. The Parent Study findings, a significant mean plasma NfL reduction of -8.9% occurred following six weeks of levacetylleucine, with a significant rebound of 9.2% during washout. This is notable for its bidirectional concordance with the clinical outcome data, in which the primary CI-CS and secondary SARA, and mDRS scores all improved during treatment and deteriorated during washout (note – this was also mirrored in the Investigator’s, Caregiver’s, and Patient’s CGI-C scores, which captured a statistically significant improvement on treatment and deterioration (worsening) during the post-treatment washout [65]. This temporal mirroring strengthens the inference that NfL changes reflect genuine biological consequences of treatment rather than random variation. Regression analyses further support this and showed a directionally consistent relationship with CI-CS though not statistically significant, the latter likely reflecting the broader, more subjective nature of that global impression scale. That the regression intercept lay at the origin for SARA is particularly meaningful, implying that NfL change and clinical change are closely coupled.

The Extension Phase data are arguably an even more compelling component of the NfL dataset. The significant progressive decline of -16.9% after one-year levacetylleucine treatment and -33.5% at two years in patients receiving continuous levacetylleucine, converging with clinical data showing 62% of EP patients achieving no worsening or improvement on mDRS against an expected trajectory of decline is consistent with a disease-modifying neuroprotective effect of levacetylleucine beyond its already demonstrated rapid improvement of neurological manifestations and functioning. Taken alongside the precedent of NfL’s acceptance as a surrogate endpoint in SOD1-ALS and analogous evidence from NPC, CLN2, CLN3, Gaucher disease, MLD, and neuronopathic mucopolysaccharidosis type II as stated in the Introduction, these findings position NfL as a biomarker that is reasonably likely to predict clinical benefit in GM2.

## Limitations

Limitations of the study include the small sample sizes, particularly in the NfL and Extension Phase analyses, the open-label design, the absence of an untreated longitudinal comparator, and the lack of adjustment for multiple comparisons. Further, missing data across visits may introduce selection bias. NfL, while sensitive, is non-specific and may be influenced by other factors. Finally, the short duration of the Parent Study limits assessment of temporal dynamics.

## Conclusion

In this study, plasma NfL decreased during levacetylleucine treatment in patients with GM2 and increased during washout, paralleling clinical outcomes. Long-term reductions in NfL further support its utility as a pharmacodynamic biomarker and demonstrated the neuroprotective, disease-modifying effect of levacetylleucine. While limited by small sample size, these findings contribute to growing evidence supporting NfL as a valid surrogate endpoint in ultra-rare neurodegenerative diseases.

## Data Availability

The datasets generated and/or analyzed during the current study are not publicly available but are available from the corresponding author on reasonable request.
